# An epitope-based malaria vaccine targeting the junctional region of circumsporozoite protein

**DOI:** 10.1038/s41541-020-00274-4

**Published:** 2021-01-21

**Authors:** Lucie Jelínková, Hugo Jhun, Allison Eaton, Nikolai Petrovsky, Fidel Zavala, Bryce Chackerian

**Affiliations:** 1 Department of Molecular Genetics and Microbiology, University of New Mexico School of Medicine, Albuquerque, New Mexico USA; 2 W. Harry Feinstone Department of Molecular Microbiology and Immunology, Malaria Research Institute, Johns Hopkins Bloomberg School of Public Health, Baltimore, MD USA; 3 Vaxine Pty Ltd, 11 Walkley Avenue, Warradale, Adelaide, SA 5046 Australia; 4 College of Medicine and Public Health, Flinders University, Adelaide, SA 5042 Australia

**Keywords:** Adjuvants, Protein vaccines, Vaccines

## Abstract

A malaria vaccine that elicits long-lasting protection and is suitable for use in endemic areas remains urgently needed. Here, we assessed the immunogenicity and prophylactic efficacy of a vaccine targeting a recently described epitope on the major surface antigen on *Plasmodium falciparum* sporozoites, circumsporozoite protein (CSP). Using a virus-like particle (VLP)-based vaccine platform technology, we developed a vaccine that targets the junctional region between the N-terminal and central repeat regions of CSP. This region is recognized by monoclonal antibodies, including mAb CIS43, that have been shown to potently prevent liver invasion in animal models. We show that CIS43 VLPs elicit high-titer and long-lived anti-CSP antibody responses in mice and is immunogenic in non-human primates. In mice, vaccine immunogenicity was enhanced by using mixed adjuvant formulations. Immunization with CIS43 VLPs conferred partial protection from malaria infection in a mouse model, and passive transfer of serum from immunized macaques also inhibited parasite liver invasion in the mouse infection model. Our findings demonstrate that a Qβ VLP-based vaccine targeting the CIS43 epitope combined with various adjuvants is highly immunogenic in mice and macaques, elicits long-lasting anti-CSP antibodies, and inhibits parasite infection in a mouse model. Thus, the CIS43 VLP vaccine is a promising pre-erythrocytic malaria vaccine candidate.

## Introduction

Malaria is a major global public health concern, causing 228 million infections and 405,000 deaths worldwide in 2018^1^. Although malaria can be caused by several species of the parasitic organism *Plasmodium*, *Plasmodium falciparum* is responsible for causing a severe form of the disease with the highest morbidity and mortality, and is one of the leading causes of death in children under 5 years old^1^. Infection is initiated when the female *Anopheles* mosquito injects sporozoites into the bloodstream of a human host. Sporozoites rapidly migrate to the liver where they transiently multiply within hepatocytes, producing merozoites. Merozoites then enter the bloodstream where they invade erythrocytes, replicate further, and cause the symptoms and pathology of malaria^2^.

Vaccines that target different stages of the malaria life cycle are under development^2^. However, only vaccines that target the pre-erythrocytic stage have potential for providing sterilizing immunity^2^. One of the primary targets of pre-erythrocytic vaccines is the major *P. falciparum* surface antigen circumsporozoite protein (*Pf*CSP)^3,4^ CSP plays a critical role in facilitating parasite invasion of hepatocytes^5^ and antibodies that target CSP can block infection^6,7^. CSP consists of an immunodominant central repeat region, which contains ~35–38 copies of an NANP motif and up to four copies of an NVDP motif, flanked by N- and C-terminal regions^8^. The sole approved malaria vaccine, RTS,S/AS01, is a recombinant protein-based vaccine developed in 1987, which comprised a large portion of CSP (consisting of 19 NANP repeats and the C-terminal region) fused to the hepatitis B surface antigen (HBsAg) and co-expressed with wild-type (WT) HBsAg to form a particulate antigen, which displays this fragment of CSP at low valency^9^. RTS,S provides only modest protection (30–50%) from clinical malaria in endemic areas and this protection has been shown to wane rapidly following immunization^10–12^.

Most candidate CSP-targeted vaccines, including RTS,S, have focused on eliciting antibodies against the central repeat region of the protein. Recently, however, potent inhibitory monoclonal antibodies (mAbs), which target a highly conserved epitope within the junctional region between the N-terminal region and the central repeat region have been identified. MGG4, CIS43, and L9 are mAbs that were isolated from human volunteers immunized with an experimental irradiated whole sporozoite vaccine (PfSPZ); these mAbs are highly effective at inhibiting liver invasion in mouse models of malaria infection^13–15^. These three mAbs bind to overlapping epitopes within the junctional region, a region of CSP that is not included in the RTS,S vaccine^4^. Interestingly, MGG4 and CIS43, but not L9, also appear to be able to weakly bind to the central NANP repeat region, suggesting the possibility that this binding promiscuity contributes to the potent neutralizing activity of this class of antibodies^16^. Although the mechanism of neutralization by these mAbs is not entirely clear, CIS43 has been shown to inhibit a proteolytic cleavage event in CSP that is critical for hepatocyte adhesion and, subsequently, invasion^14^. In addition, L9 and CIS43 have been shown, using mouse infection models, to block hepatocyte infection by preventing parasite egress from sinusoids in the liver and to induce cytotoxic death of sporozoites^15^.

The discovery of a novel site of vulnerability within CSP has several clinical implications. First, neutralizing mAbs could be used as prophylactic treatment in humans traveling to malaria endemic areas. CIS43, e.g., has recently entered Phase I trials for prophylactic prevention of clinical malaria in humans^17^. Second, these data suggest that the junctional region of CSP is a promising target for vaccine development. However, no effective vaccines targeting this region have been reported in the literature. Tan and colleagues immunized mice with a vaccine consisting of a 19 amino acid peptide encompassing the MGG4 epitope conjugated to keyhole limpet hemocyanin, but the antibodies elicited by this vaccine failed to block sporozoite invasion of hepatocytes^13^. More broadly, there are significant challenges to developing an effective liver-stage vaccine targeting malaria sporozoites. Sporozoites can reach hepatocytes in less than an hour following infection^18^, limiting the window of time in which immune responses can act. In addition, a single infected hepatocyte can seed the blood stage of the malaria life cycle, meaning that effective immunity likely must be sterilizing^2^. Thus, there is a high barrier for vaccine-mediated protection—an effective vaccine must elicit very high levels of circulating antibodies and these antibodies must be long-lived^19^.

As a potential solution, we investigated the effectiveness of an adjuvanted virus-like particle (VLP)-based vaccine targeting the CIS43 epitope. VLPs are non-infectious, self-assembling particles that are derived from viral structural proteins that can be used as standalone vaccines but also can be applied as platforms for vaccine development^20,21^. VLP-based vaccine design exploits the intrinsic ability of viral structural proteins to self-assemble into highly immunogenic, multivalent particles. These multivalent structures are effective at stimulating strong antibody responses by promoting B-cell receptor crosslinking, leading to robust and long-lasting antibody responses against diverse target antigens^20–22^. In addition, adjuvants play a key role in maximizing the ability of vaccines to induce high titer, high avidity antibodies that are durable over time. In the past, most human malaria vaccine candidates have incorporated either aluminium salts (alum) or oil emulsion adjuvants such as Montanide. However, the former have, by and large, proved insufficiently immunogenic^23^, whereas the latter have suffered from high reactogenicity^24^, raising safety concerns. In recent years, a variety of new adjuvants have become available, raising the possibility to use these to improve malaria vaccine efficacy without compromising safety. Advax adjuvants are proprietary adjuvant formulations produced by Vaxine Pty Ltd, Australia, which include novel adjuvants based on particles including those made from inulin, a plant-based polysaccharide^25,26^. These particulate adjuvants have been shown to be potent enhancers of both cellular and humoral immunity. Advax adjuvants are co-formulated with a range of immune modulators such as TLR9-active CpG oligonucleotides to achieve synergistic adjuvant effects and help steer the immune response in any desired direction.

Here we describe the development and characterization of a bacteriophage VLP-based vaccine targeting the CIS43 epitope. CIS43 VLPs elicit high-titer antibody responses against *P. falciparum* CSP in both mice and macaques, particularly in combination with Advax adjuvants, these antibody responses are highly durable, and vaccination inhibits malaria invasion of the liver in a mouse model.

## Results

### Construction and antigenicity of CIS43 VLPs

**Fig. 1:**
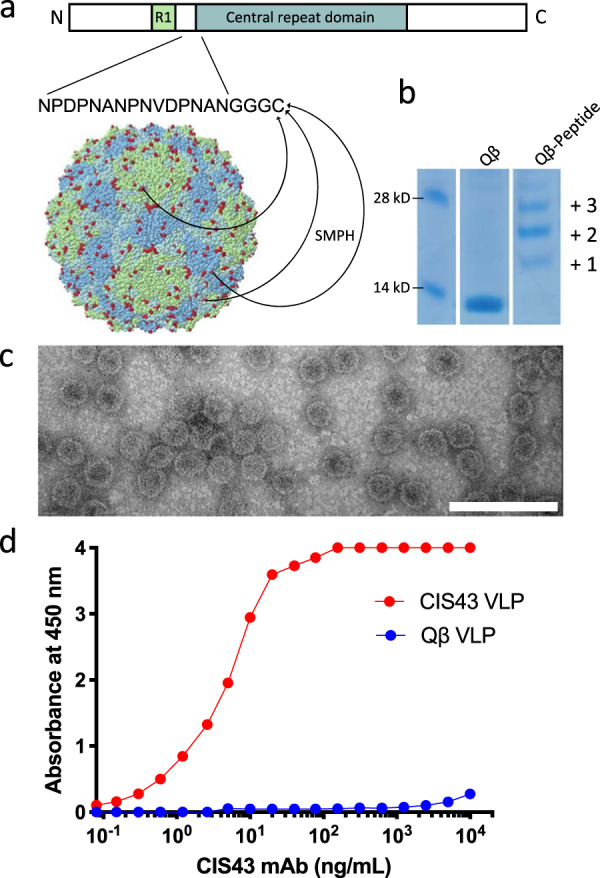
Characterization of CIS43 VLPs. **a** Schematic representation of CSP showing the location of the CIS43 epitope and the process of CIS43 VLP conjugation. A 15-amino acid peptide representing the CIS43 mAb epitope was synthesized to include a (Glycine)_3_-Cysteine linker and conjugated to surface-exposed lysine residues (shown in red) on the coat protein of Qβ bacteriophage VLPs using the bifunctional crosslinker SMPH. **b** SDS-PAGE analysis of unconjugated (center lane) or CIS43 peptide conjugated (right lane) Qβ VLPs. The ladder of bands in the CIS43 VLP lane reflect individual copies of coat protein modified with 1, 2, or more copies of the CIS43 peptide. Gel images are derived from the same experiment and were processed in parallel. Size markers are shown in the left lane. The unmodified gel is shown in Supplementary Fig. 2. **c** Transmission electron micrograph of the CIS43 VLPs. VLPs are visualized at a magnification of 200,000x. Scale bar (in white) represents 100 nm. **d** Binding of the CIS43 mAb to CIS43 VLPs (red) or wild-type (unmodified) Qβ VLPs (blue) as measured by ELISA.

The CIS43 mAb epitope was mapped to a 15-amino acid peptide at the N terminus of the repeat region of CSP (shown schematically in Fig. 1a and in more detail in Supplementary Fig. 1), spanning CSP amino acids 101–115^14^. To assess whether a vaccine targeting this epitope could elicit antibodies with CIS43-like activity, we engineered RNA bacteriophage VLPs to display the CIS43 epitope at high valency. A peptide representing CSP_101–115_ was synthesized to contain a short Gly-Gly-Gly-Cys linker sequence and then conjugated to the surface lysines on Qβ bacteriophage VLPs using a bifunctional crosslinker (shown schematically in Fig. 1a) to produce CIS43 VLPs. Conjugation efficiency was measured by SDS-polyacrylamide gel electrophoresis analysis. Successful peptide conjugation is indicated by an increase in the molecular weight of Qβ coat protein subunits, reflecting conjugation of one or more peptides to Qβ coat protein (Fig. 1b, right lane; the unmodified gel is shown in Supplementary Fig. 2). More than half of all coat protein bound two or more copies of peptide, suggesting that the particles are decorated with the peptide in a dense and multivalent fashion. We estimate that an average of 360 copies of the peptide were conjugated to each Qβ VLP. CIS43 VLPs were visualized by transmission electron microscopy (TEM), confirming their particulate, multivalent morphology (Fig. 1c). To assess the antigenicity of the CIS43 VLPs, we measured the binding of CIS43 mAb to CIS43 VLPs by enzyme-linked immunosorbent assay (ELISA). As shown in Fig. 1d, CIS43 VLPs were robustly recognized by the CIS43 mAb. These data suggest that the CIS43 epitope peptide is displayed on the Qβ particles in a manner that emulates its antigenic conformation on CSP.

### CIS43 VLPs induce high-titer antibody responses against CSP

**Fig. 2:**
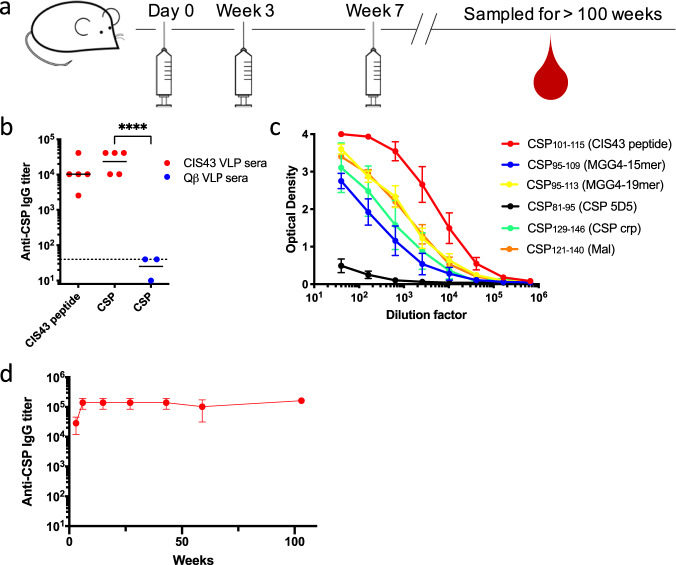
CIS43 VLPs elicit high-titer and long-lasting antibody responses against CSP. **a** CIS43 VLP immunization scheme. **b** IgG titers against CIS43 peptide or CSP were assessed after two immunizations (6 weeks after the initial vaccination). Balb/c mice were immunized with 5 µg of CIS43 VLPs (*n* = 5; red symbols) or 5 µg of WT Qβ VLPs (*n* = 3; blue symbols). Each data point represents an individual mouse and lines represent the geometric mean titers of each group. The dashed line indicates the limit of detection of the ELISA. Statistical significance between groups was determined by *t*-test (****p < 0.0001). **c** Sera from CIS43 VLP-immunized Balb/c mice were tested for binding to different CSP peptides (listed in Supplementary Fig. 1) by ELISA. Data represents the mean values from five mice immunized twice with unadjuvanted CIS43 VLPs. Error bars represent SEM. **d** Geometric mean IgG titers were determined longitudinally for over 100 weeks after the initial vaccination. It is noteworthy that three of the mice in the CIS43 VLP-vaccinated group were killed or died (at weeks 56, 60, and 60) due to health complications unrelated to vaccination and prior to the completion of the study.

We have previously shown that multivalent display of peptides on VLPs can stimulate high titer antibody responses. To assess the immunogenicity of the CIS43 VLPs in mice, Balb/c mice were intramuscularly immunized with CIS43 VLPs or, as a negative control, WT Qβ VLPs and boosted at 3 and 7 weeks (Fig. 2a). Three weeks following the final boost, antibody responses against the CIS43 peptide and full-length recombinant *P. falciparum* CSP were measured by end-point dilution ELISA. Whereas negative control sera collected from mice immunized with WT Qβ VLPs did not show reactivity to the CIS43 peptide or full-length CSP, CIS43 VLPs elicited strong anti-peptide (Fig. 2b, c) and anti-CSP antibody responses (Fig. 2b). The antibody titer to CSP was slightly higher than the anti-CIS43 peptide titer, possibly because a portion of the CIS43 epitope shares homology to the CSP repeat region^14^. Similar antibody titers were observed in C57BL/6 mice immunized with CIS43 VLPs (Supplementary Fig. 3). To demonstrate that CIS43 VLP-elicited antibodies target the same epitope as the CIS43 mAb, we tested whether sera is able to block the binding of CIS43 mAb to CSP by competition ELISA. We show that sera collected from mice immunized with CIS43 VLPs block CIS43 mAb binding (Supplementary Fig. 4). Lastly, to interrogate the binding promiscuity of elicited anti-CSP antibodies from immunized Balb/c mice, we tested the ability of sera to bind to several peptides representing epitopes within and flanking the junctional region of CSP (shown in Supplementary Fig. 1) by ELISA (these epitopes are described in refs ^13,14,27,28^). Although antisera bound most strongly to the peptide corresponding to the CIS43 epitope, we also detected cross-reactive binding to several other CSP-derived peptides (Fig. 2c), particularly those peptides that contained NPNV motifs, which are found in the junctional region, but also at the N terminus of the CSP central repeat region.

We and others have previously shown that vaccination with VLPs can elicit differentiation of long-lived plasma cells (LLPCs), resulting in stable, long-lasting antibody levels^29–31^. It is likely that long-lived circulating antibodies will be required for sustained protection from malaria infection in endemic areas, where reinfection is common. We examined the longevity of the antibody response to CIS43 VLPs in mice. Serum was collected regularly for two years after the third immunization and anti-CSP IgG titers were determined by ELISA. Remarkably, anti-CSP IgG titers were stable over this period, nearly spanning the lifetime of the mice (Fig. 2d). Together, these experiments illustrate that CIS43 VLPs elicit robust and long-lived antibody responses.

### Immunization with CIS43 VLPs protects mice from intravenous challenge with *Plasmodium*

**Fig. 3:**
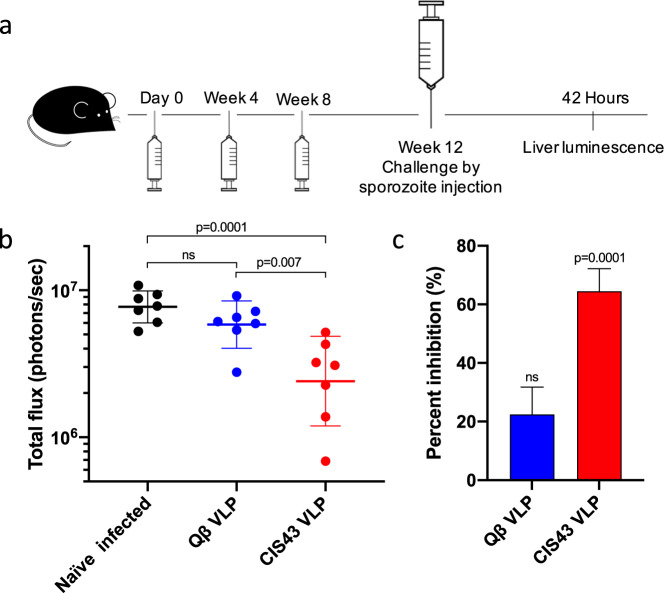
Immunization with CIS43 VLPs without exogenous adjuvant confers partial protection from *Plasmodium* infection. **a** Immunization and challenge scheme. **b** Parasite liver load (as measured by luminescence) in vaccinated or naive C57BL/6 mice following challenge by sporozoite injection. Horizontal lines represent the geometric mean luminescence of each group, error bars represent SD of the geometric mean. Baseline luminescence was measured at 10^5^ photons/s. Groups were compared statistically using one-way ANOVA followed by Dunnett’s multiple comparison test. ns; not significant. **c** Percent inhibition of liver infection, as calculated from luminescence data shown in **b**. Inhibition of infection is calculated relative to the mean signal in the naive, challenged group of mice. Mann–Whitney test was used to statistically compare each group to the naive infected control group. Error bars represent SEM.

To examine whether immunization with CIS43 VLPs confers protection from infection, we took advantage of a well-characterized mouse infection model for testing CSP-targeted vaccines. In this model, mice are challenged with transgenic *Plasmodium berghei* (*Pb*) that have been engineered to express full-length *Pf*CSP (in place of *Pb*CSP) and a luciferase reporter (*Pb-Pf*CSP-*Luc*)^32^. Forty-two hours after infection, liver-stage parasite burden can be quantitated by measuring luciferase levels. Mice were immunized three times with CIS43 VLPs or, as a negative control, WT Qβ VLPs, and then were challenged by intravenous injection of 1000 *Pb-Pf*CSP*-Luc* sporozoites four weeks following the final vaccine boost (Fig. 3a). Liver luciferase levels were compared to unimmunized (naive) mice. Mice immunized with CIS43 VLPs had a significantly reduced (~64%) liver-stage parasite burden compared to naive mice (raw data from individual mice is shown in Fig. 3b, mean inhibition data is shown in Fig. 3c). Mice immunized with WT Qβ VLPs had slightly lower (20%) parasite burden in the liver compared to naive controls, but this difference was not statistically significant (Fig. 3b, c). These data suggest that immunization with unadjuvanted CIS43 VLPs provides partial inhibition of parasite liver invasion and protection from malaria infection in a rigorous challenge mouse model.

### Advax adjuvants increase the immunogenicity of CIS43 VLPs

Immunization with CIS43 VLPs provided partial protection from in vivo challenge. Previous research showed that the protection conferred by passive antibody transfer of CIS43 mAb is dose dependent^14,15^, suggesting that higher antibody levels could potentially provide stronger protection. As a strategy to increase antibody titers elicited by CIS43 VLPs, we tested the compatibility of CIS43 VLPs with a variety of combination adjuvants. These included delta inulin polysaccharides, that have been shown to enhance both the cellular and humoral immune responses to a variety of antigens^25,26^, TLR agonists that induce strongly Th1-biased responses, and aluminum salts that induce strong Th2-biased responses. Mice were immunized three times with CIS43 VLPs in combination with either delta inulin adjuvant (Advax-1), various combination adjuvant formulations (Advax-2–5), CpG oligonucleotides alone, or without exogenous adjuvant, and then antibody levels were determined by ELISA. As shown in Supplementary Fig. 5, combination adjuvants, including those containing Toll Like Receptor (TLR) 9 or TLR7/8 agonists, increased anti-CSP antibody titers relative to unadjuvanted CIS43 VLPs or CIS43 VLPs combined with single adjuvants. All of the vaccine/adjuvant formulations induced a mean IgG1/IgG2a ratio of ~5 : 1, a ratio that was statistically indistinguishable from unadjuvanted CIS43 VLPs (Supplementary Fig. 6), and which is consistent with the previous observation that delta inulin acts by enhancing immune activation signals contained within the antigen itself^33^.

**Fig. 4:**
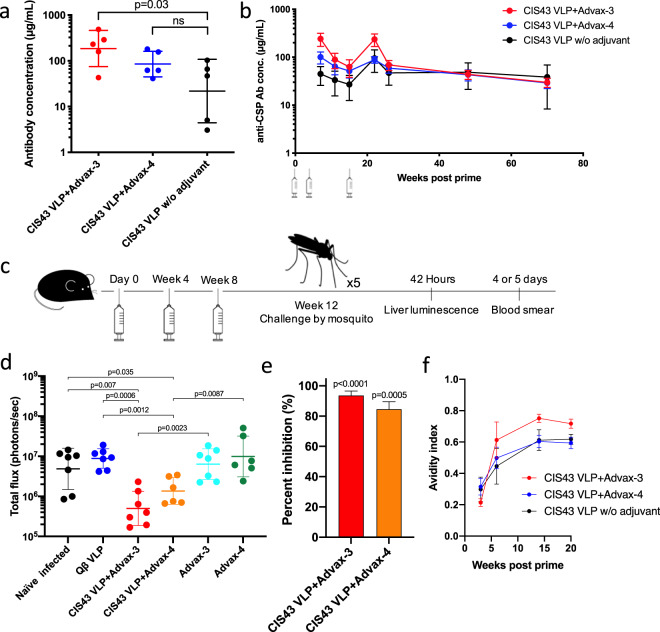
Advax adjuvants enhance both CIS43 VLP immunogenicity and protection from malaria infection. **a** Anti-CSP antibody concentrations in Balb/c mice immunized two times with CIS43 VLPs with or without Advax-3 or Advax-4 (*n* = 5/group). Groups were statistically compared to one another by unpaired *t*-test. ns; not significant. **b** Kinetics of anti-CSP antibody concentrations in Balb/c mice immunized three times with CIS43 VLPs with or without adjuvant. Mice were immunized at week 0, 4, and 18, and anti-CSP antibody concentrations were measured at various timepoints following immunization. **c** Immunization and challenge scheme. Groups of C57BL/6 mice (*n* = 6 or 7/group) were immunized three times and then challenged with five *Pb-Pf*CSP*-Luc* infected mosquitos. Liver luminescence was evaluated 42 h following mosquito challenge. **d** Parasite liver load (as measured by luminescence) in CIS43 VLP-vaccinated or control mice following mosquito challenge. Mann–Whitney test was used to statistically compare each group to the unimmunized (naive) group. **e** Percent inhibition of liver infection, as calculated from luminescence data. Inhibition of infection is calculated relative to the mean signal in the negative control groups of mice. Mann–Whitney test was used to statistically compare each group to all control groups. Error bars represent SEM. **f** Avidity index (AI) of anti-CSP antibodies elicited following immunization with CIS43 VLPs with or without adjuvant. Error bars represent SEM. Statistical comparisons between groups was determined by unpaired *t*-test using area under the curve.

Among the adjuvants that were tested, two of the combination adjuvants, Advax-3 (a more Th2-biased co-formulation of CpG55.2 oligonucleotide with aluminum hydroxide) and Advax-4 (a more Th1-biased co-formulation of delta inulin plus a TLR7/8 agonist), had the greatest boosting effect on anti-CSP titers (Supplementary Fig. 4). To more carefully measure the impact of adjuvants, we quantitated anti-CSP antibody concentrations in vaccinated mice by linear regression analysis by CSP ELISA using a standard curve generated using the mouse anti-CSP mAb 2A10^34^. After two immunizations, mice immunized with unadjuvanted CIS43 VLPs generated a mean anti-CSP IgG level of 22 µg/mL. Formulation of CIS43 VLPs with Advax-3 or Advax-4 significantly boosted anti-CSP IgG concentrations by 8.4-fold and 3.8-fold, respectively (Fig. 4a).

To evaluate the longevity of the response, we followed anti-CSP IgG responses over time. As is shown in Fig. 4b, anti-CSP antibody responses declined after the second immunization in all groups, but were restored to peak antibody levels after a third immunization, 18 weeks after the primary immunization. Antibody responses induced by unadjuvanted CIS43 VLPs were remarkably stable after the third immunization, similar to the longevity data shown in Fig. 2c. However, antibody concentrations in mice immunized with CIS43 VLPs with combination adjuvants, particularly with Advax-3, declined following the second immunization, suggesting that some of this increase in total anti-CSP antibody levels may be mediated by differentiation of B cells into short-lived plasma cells (Fig. 4b). Nevertheless, after this initial period of decline, anti-CSP IgG levels in groups immunized CIS43 VLPs plus Advax-3 or Advax-4 were largely stable.

### Vaccination with CIS43 VLPs combined with Advax-3 or Advax-4 more strongly inhibits *Plasmodium* infection in mice

To evaluate the protection elicited by CIS43 VLPs adjuvanted with Advax-3 or Advax-4, C57BL/6 mice (*n* = 6–7/group) were vaccinated with CIS43 VLPs with adjuvant, or with adjuvant alone and then were challenged with *Pb-Pf*CSP*-Luc* sporozoites. In this experiment, mice were challenged with live parasite-carrying mosquitoes to more closely recapitulate the conditions of natural infection (Fig. 4c). Mice immunized with CIS43 VLPs adjuvanted with Advax-3 or Advax-4 showed significantly lower parasite liver loads compared to the naive group, mice immunized with either adjuvant alone, or WT Qβ VLPs (Fig. 4d). Animals immunized with CIS43 VLPs adjuvanted with Advax-3 or Advax-4 showed a 90% and a 72% reduction in liver parasite loads compared to naive mice, respectively. When we compared liver loads in these two groups with an aggregate of all of the negative control groups, we measured an 93% (CIS43 VLPs with Advax-3) and a 84% (CIS43 VLPs with Advax-4) reduction (Fig. 4e). Despite these reductions in liver load, all of the mice in this study developed parasitemia, as evaluated by blood smears, by day 4 post infection, with the exception of one mouse in each of the CIS43 VLP plus Advax groups—these mice, which had the lowest liver luminescence values in their respective groups, developed parasitemia at day 5. These results demonstrate that the CIS43 VLP plus Advax vaccine is capable of stimulating high antibody responses that confers a robust, although not sterilizing, level of protection from liver infection in a mouse model.

It was previously reported in clinical trials of RTS,S/AS01 that increased antibody avidity, along with antibody concentration, correlated with increased protection from clinical malaria^19,35^. To understand how avidity of CIS43 VLP-elicited antibody changes over time and in response to added adjuvant, we evaluated the avidity index (AI) of antibodies against CSP using a chaotrope-based avidity assay. The mean AI of each group increased after each immunization, culminating in a high mean AI value (>0.5) in all of the groups following the third immunization (Fig. 4f). Among the three vaccine groups we tested, Advax-3 adjuvanted CIS43 VLPs elicited anti-CSP antibodies with the highest avidity (Fig. 4f), although this difference was not statistically significant (*p* = 0.06).

### CIS43 VLPs elicit anti-CSP antibody responses in non-human primates and these antibodies can protect mice from malaria infection upon passive transfer

**Fig. 5:**
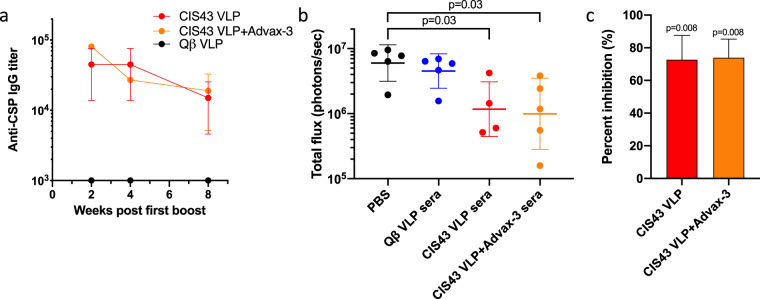
Cynomolgus monkeys immunized with CIS43 VLPs produce protective anti-CSP antibodies. **a** IgG concentrations in cynomolgus monkeys immunized at weeks 0 and 4 with CIS43 VLPs with or without Advax-3 adjuvant (*n* = 3/group). Qβ VLPs were used as a negative control. **b** Parasite liver load (as measured by luminescence) in mice (*n* = 4–5/group) that received an intravenous injection of 500 µl of sera from immunized cynomolgus monkeys. Two hours following serum transfer, mice were challenged with five *Pb-Pf*CSP*-Luc* infected mosquitoes. Mann–Whitney test was used to statistically compare each group to the group that received an intravenous injection with PBS. **c** Percent inhibition of liver infection, as calculated from luminescence data. Inhibition of infection is calculated relative to the mean signal in the control groups. Mann–Whitney test was used to statistically compare each group to all control groups. Error bars represent SEM.

CIS43 VLP immunogenicity in non-human primates was evaluated by immunizing groups (*n* = 3) of cynomolgus monkeys twice with unadjuvanted CIS43 VLPs or CIS43 VLPs adjuvanted with Advax-3. All of the macaques developed anti-CSP antibodies (Fig. 5a), but there was some heterogeneity in the responses, possibly due to the diverse ages and sizes of the animals in the study (Supplementary Table 1). Antibody levels were similar in macaques immunized with unadjuvanted CIS43 VLPs and mice immunized with CIS43 VLPs plus Advax-3. To investigate whether serum from CIS43 VLP-immunized cynomolgus monkeys could mediate protection from infection, we performed a passive transfer of monkey sera into naive C57BL/6 mice and then challenged the mice with *Pb-Pf*CSP*-Luc* by mosquito infection. Macaque sera was obtained 2 weeks after the second immunization, pooled by immunization group, and then was used to passively immunize mice. Following intravenous administration of this pooled macaque sera, mice were rested for two hours and then subjected to mosquito challenge. As before, parasite liver load was evaluated by liver luminescence. We measured a statistically significant decrease in parasite liver burden in mice passively immunized with pooled sera from macaques immunized with unadjuvanted CIS43 VLP group and Advax-3-adjuvanted CIS43 VLPs, but not using sera from macaques immunized with control WT Qβ VLPs. Sera from monkeys immunized with unadjuvanted CIS43 VLPs reduced parasite liver load by 72%, and sera from Advax-3-adjuvanted CIS43 VLPs reduced parasite liver load by 73%. Thus, these data show that non-human primates immunized with CIS43 VLPs elicit antibody responses that are capable of blocking hepatocyte invasion.

## Discussion

Development of an effective vaccine for malaria has been complicated by a number of features of the parasite, including its complex life cycle, antigenic variability of its surface proteins, and the fact that immunity to natural infection largely does not confer protection from reinfection. One of the goals of an epitope-based malaria vaccine is to direct immune responses to target vulnerable, conserved epitopes from the pathogen. Here we evaluated the efficacy of a novel vaccine targeting a highly conserved epitope^14,34,36^ within the junctional region of *P. falciparum* CSP that is recognized by several potent inhibitory mAbs, including mAb CIS43. We showed that a VLP-based vaccine that displays the CIS43 epitope elicited high-titer antibodies against CSP in both mice and non-human primates and that vaccination inhibited parasite invasion in a mouse *Plasmodium* challenge model. Passive immunization of mice with CIS43-immunized macaque sera also inhibited infection, indicating that antibodies mediate these prophylactic effects. The mechanism(s) by which antibodies induced by CIS43 VLPs provide protection is unclear, but it has been shown that the CIS43 mAb can partially inhibit proteolytic cleavage of CSP^14^, which is critical for invasion of hepatocytes^37^. CIS43 and the recently described mAb L9, an even more potent mAb that recognizes an overlapping epitope in the junctional region, can also reduce hepatocyte infection in the mouse model by preventing parasite egress from sinusoids in the liver^15^. CIS43, and other mAbs that target the junctional region of CSP^16^, also cross-reacts with the central repeat region of CSP. Antibodies that bind to the central repeat region of CSP can block infection by inhibiting sporozoite motility^18,34,38^, inducing parasite cytotoxicity^39^ and/or by enhancing immune clearance^40,41^. We showed that antibodies induced by CIS43 VLPs bound most strongly to the CIS43 epitope, but they also cross-reacted with other CSP epitopes in the junctional region and the central repeat region, raising the possibility that this binding promiscuity may contribute to their inhibitory activity. Thus, it is possible that polyreactive antibodies targeting the junctional region of CSP act through multiple mechanisms to prevent hepatocyte infection. Additional studies will be required to provide insight into the major mechanisms whereby vaccines targeting the CIS43 epitope inhibit hepatocyte invasion. Producing a vaccine that specifically targets the R1 cleavage site in the junctional region could potentially dissect the relative contributions of how antibody binding to these two regions contributes to protection.

The junctional region at the N terminus of the central repeat region of CSP contains an NPDP tetrapeptide followed by three interspersed NANP and NVDP repeats (Supplementary Fig. 1). This is followed by the central repeat region, which consists of ~35-38 NANP repeats, with a fourth NVDP tetrapeptide typically inserted after the twentieth NANP repeat. Several studies have shown that inhibitory CSP mAbs recognize epitopes derived from the joining of major and/or minor tetrapeptide repeats, including the sequences NPNA (major/major), NPNV (major/minor), and DPNA (minor/major)^15,42,43^. CIS43, e.g., strongly binds to DPNA-containing sequences from CSP^15^. In contrast, L9, which is likely the most potent anti-CSP mAb described to date (~4-fold more potent than CIS43), prefers binding to the NPNV motifs that form at the junctions of interspersed NANP and NVDP repeats^15^. The CIS43 epitope-derived peptide that we utilized in this vaccine study contains two DPNA motifs and a single NPNV motif, and antibodies raised by immunization with CIS43 VLPs bound most strongly to peptides containing DPNA motifs (Fig. 2c). Given the potency of the L9 mAb, it would be interesting to test whether a VLP-based vaccine that preferentially induced antibodies targeting the NPNV motif could induce more strongly protective antibody responses. Such a vaccine could potentially be combined with the CIS43 epitope-targeted vaccine described in this study.

The ability to elicit durable antibody responses through the induction of long-lived antibody secreting plasma cells is likely to be particularly important for providing protection from malaria infection. Liver infection occurs within a few minutes or hours of parasite exposure and once *Plasmodium* invades hepatocytes it is not accessible to anti-sporozoite antibodies^44^. Thus, circulating antibodies, but not reactivated memory B cells, are likely to be a critical mediator of effective immunity to malaria sporozoites. Indeed, one of the primary shortcomings of RTS,S is that antibody levels decrease rapidly following immunization^45^. Here we showed that CIS43 VLPs not only elicited high titer antibody responses against CSP, but that these antibodies were extremely durable. Over the span of a nearly 2-year period following vaccination, mouse anti-CSP antibody titers did not decline. These data add to a growing body of literature demonstrating that the ability to induce LLPCs is a feature of vaccine antigens, such as VLPs, with highly multivalent structures^29–31^. Our study provides evidence that use of a multivalent vaccine technology can effectively elicit durable antibody responses against CSP.

There have been several other efforts to develop multivalent vaccines targeting CSP. R21 is a RTS,S-like vaccine in which the central repeat region of CSP is displayed at much higher density on recombinant HBsAg particles than RTS,S. R21 elicits potent immune responses against CSP^46^ and is currently in Phase 1/2a clinical trials. Several groups have applied a similar approach using bacteriophage VLP-based vaccines to display full-length CSP at high valency and have shown that these vaccines are immunogenic in mice^47^ and can confer protection following transgenic parasite challenge^48^. Whitacre et al.^27^ developed epitope-targeted vaccines that employ woodchuck hepatitis virus core antigen (WHcAg) VLPs to display B- and T-cell epitopes from repeat and non-repeat regions of CSP. VLPs displaying repeat, but not non-repeat, epitopes were capable of eliciting sterilizing immunity that prevented blood-stage infection in a mouse model. These latter challenge studies utilized a transgenic *Pb* CSP that only contained the repeat region of *Pf*CSP (unlike our studies in which transgenic *Pb* carried full-length *Pf*CSP). Thus, it is difficult to compare the relative protection provided by WHcAg-based VLPs versus the CIS43 VLPs described in this study. Nevertheless, a common finding is that VLP display of CSP epitopes by multiple platform technologies is effective at eliciting high-titer antibody responses against CSP.

We have previously shown that bacteriophage VLPs can induce strong antibody responses in the absence of exogenous adjuvants; co-administration with most standard adjuvant formulations only resulted in modest boosts in antibody titer^49^. This is due to the repetitive structure of VLPs, which strongly stimulates B cells^21,50^, but also the fact that bacteriophage VLPs encapsidate single stranded RNA, which can serve as a natural endogenous adjuvant. Here we showed that combining CIS43 VLPs with two different combination adjuvants, one combining a TLR9-active oligonucleotide (CpG55.2) with aluminum hydroxide (Advax-3) and the other combining delta inulin with a TLR7/8 agonist (Advax-4) substantially increased immunogenicity in mice relative to unadjuvanted CIS43 VLPs, although the addition of adjuvant did not boost antibody titers in non-human primates. Importantly, Advax adjuvants have been shown to be safe and efficacious in multiple human vaccine trials^26^ and recently the US Food and Drug Administration approved an IND application for an enhanced seasonal influenza vaccine containing an Advax-CpG formulation. Although the use of Advax adjuvants increased peak antibody concentrations, one limitation is that these levels rapidly declined to concentrations that were similar to the levels observed in mice immunized with unadjuvanted CIS43 VLPs.

Although the monoclonal-like response conferred by the single epitope displaying CIS43 VLPs did not confer sterilizing immunity against malaria in a mouse challenge model, the degree of protection elicited by this vaccine highlights the importance of this particular epitope for future clinical applications, and the potential efficacy of additional VLP-based vaccines that target critical epitopes within the junctional region of CSP. Further development of CIS43 VLPs, in combination with vaccines that induce antibody and/or T-cell responses against other liver- or blood-stage malarial antigens, could increase the breadth and potency of protection.

## Methods

### Production and characterization of CIS43 epitope displaying bacteriophage Qβ VLPs

Qβ bacteriophage VLPs were produced in *Escherichia coli* using methods previously described for the production of bacteriophage PP7 VLPs^51^. With the exception of the VLPs used in preliminary mouse immunogenicity studies, all CIS43 VLPs and WT Qβ VLPs were depleted of endotoxin by three rounds of phase separation using Triton X-114 (Sigma-Aldrich), as described in ref. ^52^. The fifteen amino acid CIS43 epitope peptide was synthesized (GenScript) and modified to contain a C-terminal *gly-gly-gly-cys* linker sequence (NPDPNANPNVDPNAN*GGGC*). The peptide was conjugated directly to surface lysines on Qβ bacteriophage VLPs using the bidirectional crosslinker succinimidyl 6-[(β-maleimidopropionamido) hexanoate] (SMPH; Thermo Fisher Scientific) as previously described^51^. The efficiency of conjugation was assessed by gel electrophoresis using a 10% SDS denaturing polyacrylamide gel. Peptide conjugation results in a mobility shift of the Qβ coat protein due to an increase in molecular weight. The percentage of coat protein with zero, one, two, or more attached peptides was determined using ImageJ software and used to calculate average peptide density per VLP. Presence of the CIS43 peptide on CIS43 VLPs was confirmed by ELISA. Briefly, 250 ng of VLPs were used to coat wells of an ELISA plate. Wells were probed with dilutions of mAb CIS43 (generously provided by Robert Seder, NIH Vaccine Research Center), followed by a 1 : 4000 dilution of horseradish peroxidase (HRP) labeled goat anti-human IgG (Jackson Immunoresearch). The reaction was developed using 3,3′,5,5′-tetramethylbenzidine (TMB) substrate (Thermo Fisher Scientific) and stopped using 1% HCl. Reactivity of the CIS43 mAb for the CIS43 VLPs was determined by measuring optical density at 450 nm (OD_450_) using an AccuSkan plate reader (Fisher Scientific). For TEM analysis, VLPs were coated onto carbon support film on copper grids and stained with 1% uranyl acetate for 1 min. VLPs were imaged with Hitachi HT7700 TEM with an AMT XR16M digital camera.

### Ethics statement for animal studies

All animal research complied with the Institutional Animal Care and Use Committee of the University of New Mexico School of Medicine (Approved protocol number: 19-200870-HSC), Johns Hopkins University (Approved protocol permit number: MO18H419). AlphaGenesis, Inc. adheres to the NIH Office of Laboratory Animal Welfare standards (OLAW welfare assurance #A3645-01) and all guidelines of AGI Ethics and Compliance Program were followed.

### Mouse immunization studies

Groups of 4-week-old female Balb/c mice (obtained from the Jackson Laboratory) were immunized intramuscularly with 5 μg of CIS43 VLPs or control (unmodified) Qβ VLPs. Mice were typically boosted twice after the initial prime, at 3- or 4-week intervals. Some vaccinations were performed using proprietary adjuvants generously provided by Vaxine Ptd Ltd. These adjuvants include Advax-1 (delta inulin alone), Advax-2 (delta inulin plus a TLR9 agonist), Advax-3 (CpG55.2 [a TLR9 agonist described below] plus aluminum hydroxide), Advax-4 (delta inulin plus a TLR7/8 agonist), and Advax-5 (delta inulin plus a TLR4 agonist). In these experiments, mice were intramuscularly immunized with 5 μg VLPs in combination with 20 μl the following: Advax (50 mg/mL), Advax-2 (50 mg/mL), Advax-3 (5 mg/mL), Advax-4 (50 mg/mL), Advax-5 (50 mg/mL), and CpG55.2 (0.5 mg/mL)—a synthetic Class B CpG oligonucleotide adjuvant that is dually active on both mouse and human TLR9 (unpublished data). An additional group of mice received 5 µg of unadjuvanted CIS43 VLPs. In these experiments, mice were boosted four weeks after the prime and selected groups received a second boost at 3 months. In each mouse experiment, serum samples were collected prior to each boost and, in some cases, at additional later timepoints following the final boost.

### Cynomolgus monkey immunization studies

Groups (*n* = 3/group) of male and female cynomolgus monkeys (*Macaca fascicularis*) of varying ages (9.9–17 years) and body weights (4.00–13.30 kg) were immunized with 100 µg of unadjuvanted CIS43 VLPs, or the same dose in combination with 0.5 mg of Advax-3. One month after the prime, animals were boosted with 50 µg of VLPs with or without Advax-3. A negative control group received similar doses of unmodified WT Qβ VLPs. Serum was collected at the initial immunization and at 2-week intervals thereafter.

### Quantitating antibody responses

Serum antibodies against full-length CSP were detected by ELISA using recombinant CSP expressed in *Pseudomonas fluorescens*^53^ (and generously provided by Gabriel Gutierrez at Leidos, Inc.) as the coating antigen. Immulon 2 plates (Thermo Scientific) were coated with 250 ng of CSP in 50 μl phosphate-buffered saline (PBS) and incubated at 4 °C overnight. Following incubation, wells were blocked with PBS–0.5% milk for 2 h at room temperature. Sera isolated from immunized animals were serially diluted in PBS–0.5% milk and applied to wells and incubated at room temperature for 2.5 h. Reactivity to the target antigen was detected using HRP-labeled goat anti-mouse IgG or, for macaque sera, HRP-labeled goat anti-human IgG (both Jackson Immunoresearch, diluted 1 : 4000). Reactivity was determined using TMB substrate as described above. End-point dilution titer was defined as the greatest sera dilution that yielded an OD_450_ > 2-fold over background. For mouse sera, anti-CSP antibody concentrations were also quantitated by ELISA by generating a standard curve using known concentrations of the anti-CSP mouse mAb 2A10^7,31,48^. For isotype analysis, mouse serum samples were diluted 1 : 10,000, and isotype-specific responses were determined using HRP-labeled rat anti-mouse IgG1 or IgG2a (Zymed, diluted 1 : 4000).

For peptide ELISAs, Immulon 2 plates were coated with 500 ng streptavidin (Invitrogen) for 2 h at 37 °C. Following washing, SMPH was added to wells at 1 μg/well and incubated for 1 h at room temperature. Specific peptides were added to the wells at 1 μg/well and incubated overnight at 4 °C. Wells were then incubated with dilutions of mouse sera and binding was detected as described above.

Competition ELISAs were performed by using the CSP ELISA protocol, as described above, with the following modifications: after mouse serum was added to the plate, 40 ng of the human mAb CIS43 (at a final concentration of 400 ng/mL) was added to each well and incubated for 30 min. CIS43 mAb binding to CSP was detected using HRP-labeled goat anti-human IgG at a 1 : 4000 dilution.

### Measurement of antibody avidity

The AI of anti-CSP antibodies was evaluated using an ELISA-based chaotrope avidity assay^54^. This protocol followed the standard ELISA (described above), except that following serum incubation wells were treated with 6 M urea for 10 min. Serum dilutions corresponding to comparable OD value for each group were chosen to control for differences in antibody concentrations. The AI was calculated as the ratio of the ELISA absorbance value of 6 M urea-treated wells (A_6M UREA_) to control wells incubated in water (*A*_c_); AI = *A*_6M UREA_/*A*_c_. Multiple dilutions of sera were analyzed and all samples were tested in duplicate.

### Mouse Pb-PfCSP-Luc sporozoite challenge

Challenge studies were performed using female 6–8-week-old C57BL/6 mice. Mice (typically *n* = 7/group) were immunized intramuscularly with 5 µg of CIS43 VLPs with or without adjuvant three times at 3-week intervals. Separate control groups were immunized with unmodified WT Qβ VLPs, PBS, or adjuvant alone. Each immunogen was blinded to minimize the potential for bias in animal handling during the challenge portion of the study. Serum was collected following the third immunization.

Mice were challenged using transgenic *P. berghei* sporozoites engineered to express luciferase and full-length *P. falciparum* CSP in place of *P. berghei* CSP (denoted as *Pb-Pf*CSP*-Luc*)^32^. Mice were challenged directly using sporozoites or by using infected mosquitos. For the sporozoite challenge, *Pb-Pf*CSP*-Luc* sporozoites were freshly harvested from female *Anopheles stephensi* salivary glands. One thousand sporozoites in 200 µl Hank’s buffered salt solution/2% fetal calf serum were intravenously injected into immunized and naive mice. Forty-two hours post challenge, mice were intraperitoneally injected with 100 µl d-luciferin (30 mg/ml) and anesthetized. Liver luminescence was assessed by IVIS Spectrum Imaging System (Perkin Elmer). For mosquito challenges, *A. stephensi* mosquitos were infected by blood feeding on *Pb-Pf*CSP*-Luc* infected mice. Prior to challenge, mice were anesthetized with 2% Avertin and then exposed to five mosquitos for a blood meal. Following feeding, mosquitos positive for a blood meal were counted. Liver luminescence was assessed 42 h post challenge, as described above. Five days later, blood smears were evaluated by Giemsa staining for parasitemia.

### Passive transfer study

Cynomolgus monkey sera was pooled within each group, heat inactivated for 30 min at 56 °C, and filtered through a 0.45 micron filter to remove aggregates. Sera (500 µl; or PBS as a control) was then passively transferred into each mouse (*n* = 4–5 mice/group) intravenously via the tail vein by slow injection. Two hours following serum transfer, mice were challenged by *A. stephensi* mosquito bite, as described above. Liver luminescence was evaluated 42 h post challenge, and parasitemia was evaluated by blood smears 4 days later, as described above.

### Statistics

All statistical analyses of data were performed using GraphPad Prism 8 using two-sided tests. For percent inhibition calculations (Figs. 3c, 4e, and 5c), the liver luminescence values of individual vaccinate mice was divided by the mean of mice in negative control groups. Background luminescence levels (10^5^) were subtracted from all values.

### Reporting summary

Further information on research design is available in the Nature Research Reporting Summary linked to this article.

## Supplementary information

Supplemental Information

Reporting Summary

## Data Availability

The datasets used and/or analyzed in the current study are available from the corresponding author upon reasonable request.
