# Supplementary material for: An epitope-based malaria vaccine targeting the junctional region of circumsporozoite protein
Source: NPJ Vaccines. 2021 Jan 21;6:13. doi: 10.1038/s41541-020-00274-4 (PMC7820318; doi:10.1038/s41541-020-00274-4)
Supplement: Supplementary file 1 — Supplemental Information [file 41541_2020_274_MOESM1_ESM.pdf]

Supporting information:

```
  80-NEDNEKLRKP KHKKLKQPAD GNPDPNANPN VDPNANPNVD PNANPNVDPN ANPNANPNAN PNANPNANP-148

5D5:   EDNEKLRKP KHKKLK
MGG4 (15mer):        KQPAD GNPDPNANPN
MGG4 (19mer):        KQPAD GNPDPNANPN VDPN
CIS43:                      NPDPNANPN VDPNAN
MAL:                                             NANPNVDPN ANPNANPNAN P
Central Repeat Peptide:                                  N ANPNANPNAN PNANPNA
```

Supplementary Figure 1. Sequence of the CSP junctional region and the peptides used in this study. Colors indicate the N-terminal region (blue), R1 cleavage site (red), and repeat region (black). All peptides were also synthesized to contain a C-terminal -*GGGC* sequence to facilitate crosslinking.

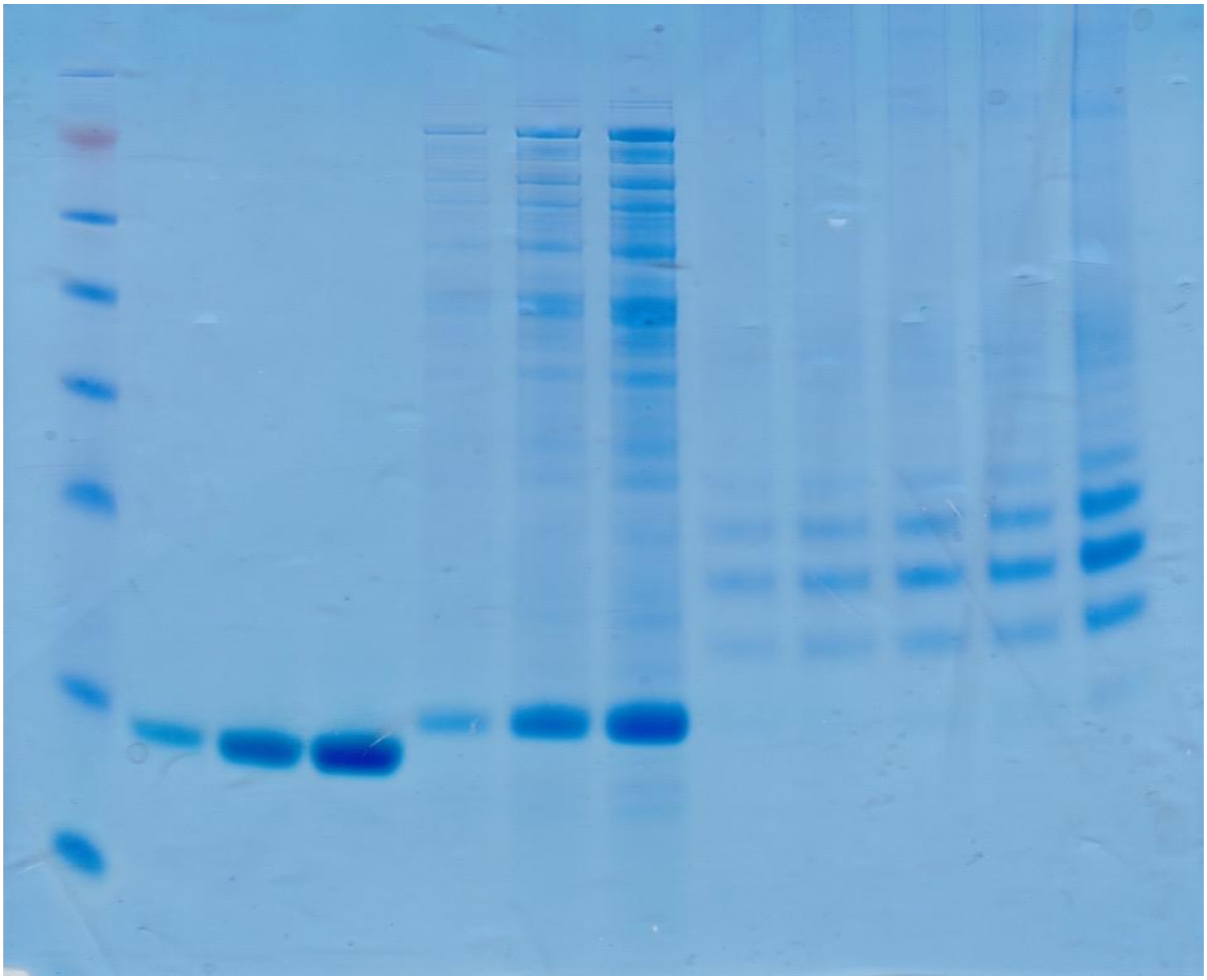

Supplementary Figure 2. Unmodified gel used as a source of the data shown in Figure 1b

Lane 1 (left) contains molecular weight markers (Invitrogen SeeBlue Plus2 Prestained Markers). Lanes 2-4 contain different amounts of Hen Egg Lysozyme (Gold Biotechnology). Lanes 5-7 contain different amounts of unconjugated Qß VLPs. Lanes 8-12 contain different amounts of CIS43 peptide conjugated VLPs. Portions of lanes 1, 6, and 11 are shown in Figure 1b.

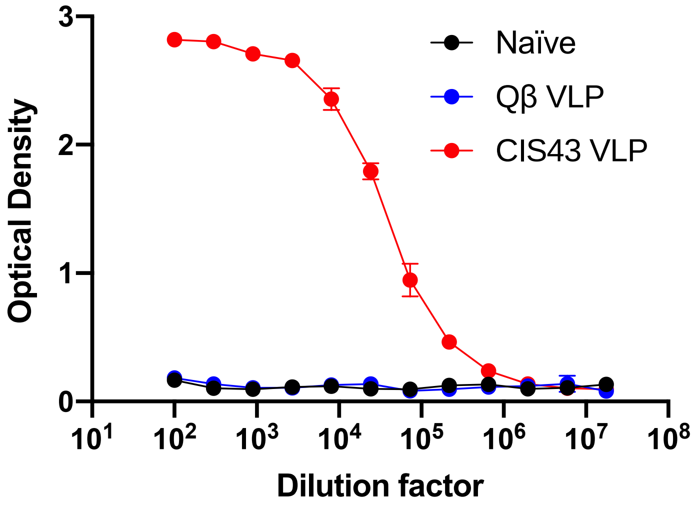

Supplementary Figure 3. CIS43 VLPs induce anti-CSP IgG antibodies in C57BL6 mice. Sera was collected from mice (n=7/group) immunized three times with CIS43 VLPs (red symbols), Qß VLPs (blue symbols), or from naïve mice (black symbols). Sera was pooled and tested (in duplicate) for reactivity against full-length CSP by ELISA. Error bars represent SD.

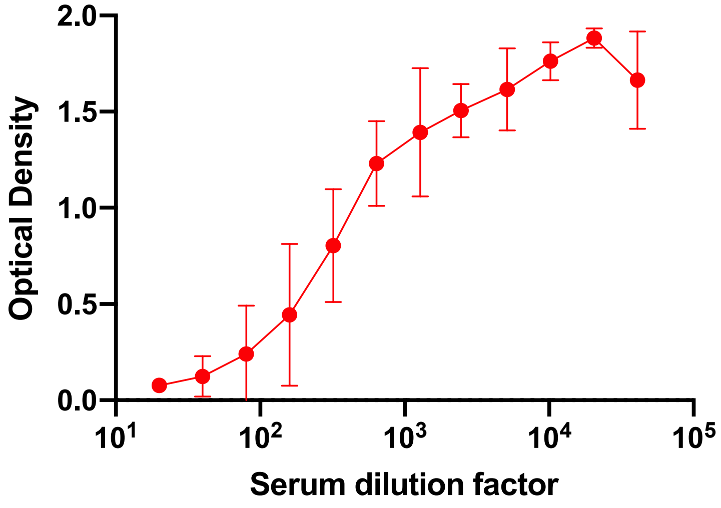

Supplementary Figure 4. CIS43 VLP mouse sera block the binding of CIS43 mAb to CSP. Sera collected from mice immunized with CIS43 VLP adjuvanted with Advax-3 (n=5) were tested for inhibition of CIS43 mAb binding to CSP. Error bars represent SD.

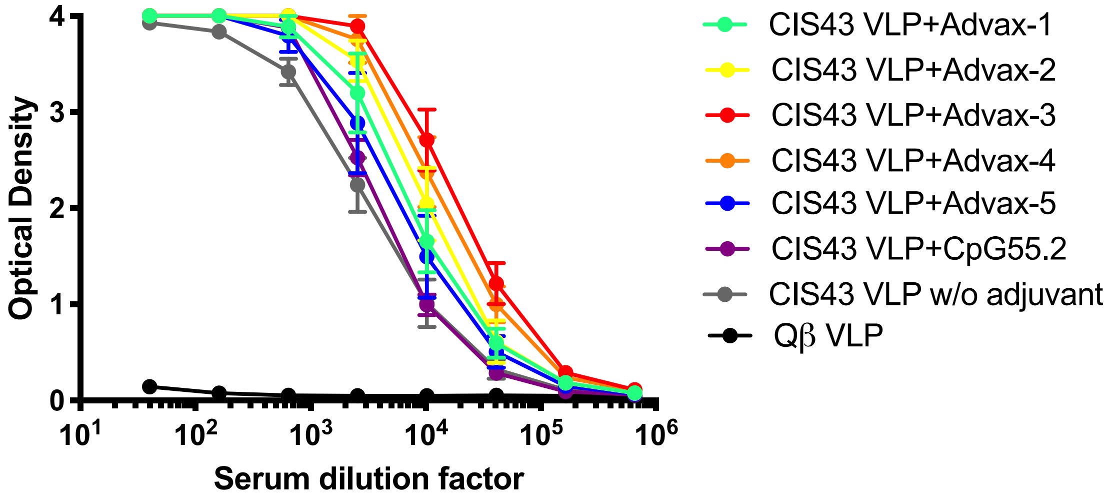

Supplementary Figure 5. Advax adjuvants enhance anti-CSP IgG response to CIS43 VLP immunization.

Groups of Balb/c mice (n = 5) were immunized with two doses of CIS43 VLPs adjuvated with different Advax adjuvant formulations or CpG oligonucleotides. Unadjuvanted CIS43 VLPs and WT Qß VLPs were used as controls. Serum was collected three weeks following the second immunization. Error bars represent SEM.

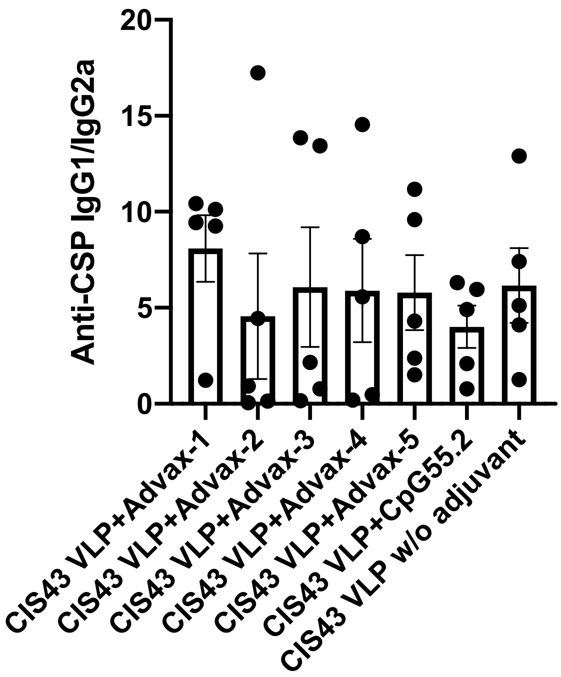

Supplementary Figure 6. Ratio of detected IgG1 to IgG2A in serum of Balb/c mice immunized with adjuvanted CIS43 VLPs.

No statistical differences were detected in anti-CSP IgG isotypes in mice immunized with CIS43 VLPs adjuvanted with Advax-1, Advax-2, Advax-3, Advax-4, Advax-5, or CpG55.2 compared to those immunized with unadjuvanted CIS43 VLPs (n=5/group). Dots represent the IgG1/IgG2a ratio of individual mice. Error bars represent SEM.

Vaccine formulation	Sex	Age (years)	Weight (kg)
CIS43 VLPs w/o adjuvant	M	16.9	13.3
	F	13.6	8
	F	15.8	4.2
Qß VLPs	M	13.5	3.35
	F	14.5	5.2
	F	16.9	5.8
CIS43 VLPs+Advax-3	F	9.9	6.9
	F	17	4
	F	13.5	5

Supplementary Table 1. Demographic information of cynomolgus monkeys in the three vaccine groups.
