# Supplementary material for: An epitope-based malaria vaccine targeting the junctional region of circumsporozoite protein
Source: NPJ Vaccines. 2021 Jan 21;6:13. doi: 10.1038/s41541-020-00274-4 (PMC7820318; doi:10.1038/s41541-020-00274-4)
Supplement: Supplementary file 2 — Reporting Summary [file 41541_2020_274_MOESM2_ESM.pdf]

nature research

Corresponding author(s): Chackerian

Last updated by author(s): Dec 4, 2020

Reporting Summary

Nature Research wishes to improve the reproducibility of the work that we publish. This form provides structure for consistency and transparency in reporting. For further information on Nature Research policies, see our Editorial Policies and the Editorial Policy Checklist.

Statistics

For all statistical analyses, confirm that the following items are present in the figure legend, table legend, main text, or Methods section.

n/a	Confirmed	
☐	☒	The exact sample size (n) for each experimental group/condition, given as a discrete number and unit of measurement
☐	☒	A statement on whether measurements were taken from distinct samples or whether the same sample was measured repeatedly
☐	☒	The statistical test(s) used AND whether they are one- or two-sided<br>*Only common tests should be described solely by name; describe more complex techniques in the Methods section.*
☒	☐	A description of all covariates tested
☒	☐	A description of any assumptions or corrections, such as tests of normality and adjustment for multiple comparisons
☒	☐	A full description of the statistical parameters including central tendency (e.g. means) or other basic estimates (e.g. regression coefficient) AND variation (e.g. standard deviation) or associated estimates of uncertainty (e.g. confidence intervals)
☒	☐	For null hypothesis testing, the test statistic (e.g. F, t, r) with confidence intervals, effect sizes, degrees of freedom and P value noted<br>*Give P values as exact values whenever suitable.*
☒	☐	For Bayesian analysis, information on the choice of priors and Markov chain Monte Carlo settings
☒	☐	For hierarchical and complex designs, identification of the appropriate level for tests and full reporting of outcomes
☒	☐	Estimates of effect sizes (e.g. Cohen's d, Pearson's r), indicating how they were calculated

Our web collection on statistics for biologists contains articles on many of the points above.

Software and code

Policy information about availability of computer code

Data collection	n/a
Data analysis	Data analysis was performed using GraphPad Prism 8.

For manuscripts utilizing custom algorithms or software that are central to the research but not yet described in published literature, software must be made available to editors and reviewers. We strongly encourage code deposition in a community repository (e.g. GitHub). See the Nature Research guidelines for submitting code & software for further information.

Data

Policy information about availability of data

All manuscripts must include a data availability statement. This statement should provide the following information, where applicable:

- Accession codes, unique identifiers, or web links for publicly available datasets
- A list of figures that have associated raw data
- A description of any restrictions on data availability

The datasets used and/or analyzed in the current study are available from the
corresponding author upon reasonable request.
The datasets used and/or analyzed in the current study are available from the
corresponding author upon reasonable request.
All data used and/or analyzed in this study are available from the corresponding author upon reasonable request.

Field-specific reporting

Please select the one below that is the best fit for your research. If you are not sure, read the appropriate sections before making your selection.

☒ Life sciences ☐ Behavioural & social sciences ☐ Ecological, evolutionary & environmental sciences

For a reference copy of the document with all sections, see nature.com/documents/nr-reporting-summary-flat.pdf

Life sciences study design

All studies must disclose on these points even when the disclosure is negative.

Sample size	sample size was determined using power analysis.
Data exclusions	no data was excluded
Replication	All data from animal experiments are reported in the manuscript. For in vitro assays, experiments were performed in duplicate or by using multiple dilutions of a sample to ensure reproducibility.
Randomization	Mice were randomized into experimental groups
Blinding	For plasmodium challenge experiments, investigators were blinded as to the vaccines or sera tested.

Reporting for specific materials, systems and methods

We require information from authors about some types of materials, experimental systems and methods used in many studies. Here, indicate whether each material, system or method listed is relevant to your study. If you are not sure if a list item applies to your research, read the appropriate section before selecting a response.

Materials & experimental systems

n/a	Involved in the study	
☐	☒	Antibodies
☒	☐	Eukaryotic cell lines
☒	☐	Palaeontology and archaeology
☐	☒	Animals and other organisms
☒	☐	Human research participants
☒	☐	Clinical data
☒	☐	Dual use research of concern

Methods

n/a	Involved in the study	
☒	☐	ChIP-seq
☒	☐	Flow cytometry
☒	☐	MRI-based neuroimaging

Antibodies

Antibodies used	mAb CIS43 was obtained from Robert Seder at the NIH Vaccine Research Center, mAb 2A10 was produced by the Zavala laboratory
Validation	Specificity for CSP was validated by ELISA

Animals and other organisms

Policy information about studies involving animals; ARRIVE guidelines recommended for reporting animal research

Laboratory animals	For immunization studies, 4-week old female Balb/c mice were used. Plasmodium challenge studies utilized 6-8 week old female C57BL/6 mice. NHP immunization studies were performed using 9.9-17 year old cynomolgus monkeys of both sexes.
Wild animals	n/a
Field-collected samples	n/a
Ethics oversight	All animal research complied with the Institutional Animal Care and Use Committee of the University of New Mexico School of Medicine (Approved protocol #: 19-200870-HSC), Johns Hopkins University (Approved protocol permit #: MO18H419). AlphaGenesis Inc. adheres to the NIH Office of Laboratory Animal Welfare standards (OLAW welfare assurance #A3645-01) and all guidelines of AGI Ethics and Compliance Program were followed.

Note that full information on the approval of the study protocol must also be provided in the manuscript.
